# Editorial: Measurement in health psychology, volume II

**DOI:** 10.3389/fpsyg.2025.1658599

**Published:** 2025-08-13

**Authors:** Victor Zaia, Giulia Casu, Antonio de Padua Serafim, Paola Gremigni

**Affiliations:** ^1^Postgraduate Program in Health Sciences, Centro Universitário FMABC, Santo André, Brazil; ^2^Department of Psychology, University of Bologna, Bologna, Italy; ^3^Institute of Psychology, University of São Paulo, São Paulo, Brazil

**Keywords:** health psychology, psychometrics, measurement, validation, scale development

## Introduction

Disruptive events such as the COVID-19 pandemic, climate-related disasters, and escalating violence across diverse sociocultural contexts have had profound repercussions on the mental health of populations. In this context, Health Psychology is a multidisciplinary field that integrates perspectives from biology, behavioral science, and the social sciences to examine how these domains interact to influence health and disease. This field employs theoretical models, evidence-based methodologies, and clinical expertise to assess, manage, and prevent a broad spectrum of conditions impacting both physical and mental health. The core objectives of Health Psychology include the promotion of health, the prevention of illness, the facilitation of treatment and recovery processes, and the identification of key biological and psychosocial determinants associated with health outcomes, disease onset, and functional impairments (Abraham et al., 2024; APA, 2025).

Measurement plays a pivotal role in Health Psychology, underpinning the rigorous assessment of psychological constructs, health-related behaviors, and individual responses to illness and healthcare. Since its emergence as a distinct discipline, Health Psychology has prioritized the design and evaluation of interventions aimed at enhancing health outcomes—by mitigating risk behaviors, promoting healthy lifestyles, and supporting the management of chronic conditions. The effectiveness and scientific validity of these interventions depend on the use of psychometrically sound instruments capable of capturing the complex psychological and social dimensions of health and disease (Freedland, 2021; Scholz, 2019). Grounded in the biopsychosocial model (Bolton, 2022)—a foundational theoretical framework in Health Psychology—measurement practices play a critical role in elucidating the determinants and consequences that shape both physical and mental health.

It is in this context that, and 3 years after the publication of the inaugural issue of *Measurement in health psychology, volume II* (Casu et al., 2022), that we are pleased to introduce the second volume of this Research Topic, reflecting its significant scientific impact and the sustained demand for methodological innovation. This initiative underscores the persistent need for methodological advancement in a field of psychology that is fundamentally grounded in empirical evidence. Health Psychology equires assessment tools that are not only psychometrically robust—exhibiting strong validity, reliability, and sensitivity—but also conceptually rigorous and contextually relevant. Within this framework, the Research Topic *Measurement in health psychology, volume II* was conceived to provide an updated and integrative overview of contemporary approaches to psychological measurement in health-related domains. This second volume brings together contributions that advance theoretical frameworks, refine measurement techniques, and address emerging challenges in the development and validation of psychological instruments. These contributions are relevant across both research and applied settings. Our aim is to foster the production of reliable, valid, and actionable data in Health Psychology, with meaningful implications for scientific research, clinical practice, and health policy formulation.

This Research Topic comprises 15 peer-reviewed articles authored by 66 scholars from diverse countries and regions. The included studies focus on the development, validation, and refinement of self-report instruments designed to assess psychological, behavioral, and socio-environmental factors that influence health trajectories and the course of illness.

## Measurement in clinical and healthcare contexts

Eight articles within this Research Topic focus on the development and validation of measurement tools tailored for clinical and healthcare settings. These contributions address screening instruments, outcome assessments, and context-specific health challenges.

Zaia et al. introduced the *Infertility Concern Questionnaire* (ICQ), validated among Brazilian couples undergoing fertility treatment. The ICQ captures two core dimensions—parental desire and communication about infertility—within a dyadic framework. Confirmatory factor analysis (CFA) supported a two-factor structure, and measurement invariance across partners confirmed its applicability to both sexes, offering preliminary evidence of its validity in fertility care.

In the field of adolescent mental health, Jiang et al. validated the *Depression Anxiety Stress Scale for Youth* (DASS-Y) in a sample of Chinese adolescents. The three-factor structure (depression, anxiety, stress) was confirmed via CFA, and the scale demonstrated strong internal consistency, test-retest reliability, and both convergent and criterion-related validity, supporting its use in routine school-based mental health screening.

Tai et al. compared the EQ-5D-3L and EQ-5D-5L in patients with knee osteoarthritis following unicompartmental knee arthroplasty. Their findings demonstrated the superior validity, reliability, and discriminant ability of the EQ-5D-5L, supporting its use for a more precise and reliable assessment of quality of life 1 year post-surgery in this population.

Ráczová et al. evaluated the *Developmental monitoring and screening method for the 11th check-up in primary care* (S-PMV11). This is a screening tool designed for monitoring the developmental difficulties based on parental assessment in primary pediatric care in Slovakia. Using Rasch modeling and Guttman scaling, the study confirmed unidimensionality and strong scalability, highlighting the tool's utility for early detection of developmental problems in pediatric care.

Tan et al. validated the Chinese version of the *Somatosensory Amplification Scale* (SSAS-C), which measures the tendency to perceive somatic sensations as unusually intense and alarming. The one-factor model showed good fit, and the SSAS-C was found to mediate the relationship between alexithymia and somatization, underscoring its relevance in psychosomatic assessment.

Santibáñez-Palma et al. developed and validated the *Environmental Confinement Stressors Scal*e (ECSS-20) designed to assess the psychological impact of COVID-19-related confinement. The scale comprises four domains—economic stressors, social activity restrictions, habitability, and exposure to virtual media—and demonstrated strong factorial validity, internal consistency, and gender invariance, supporting its use in post-pandemic health surveillance.

Ay et al., validated the Turkish version of the *MisoQuest*, a questionnaire assessing sound sensitivity and its emotional consequences. Results indicated that anxiety significantly mediated the relationship between misophonia and quality of life, underscoring the clinical relevance of recognizing this often-overlooked condition and its broader implications for psychological wellbeing in the context of clinical assessment.

Finally, Liu et al. validated the *Oncology Nurses Health Behaviors Determinants Scale* (HBDS-ON) in China. The scale assesses six determinants of health behavior among nurses—perceived threat, benefits, barriers, self-efficacy, cues to action, and access to personal protective equipment. It demonstrated strong internal consistency for the six-factor model, offering a promising tool for enhancing occupational health interventions in clinical settings.

## Psychological wellbeing and quality of life

Four studies included in this Research Topic focused on the assessment of constructs related to psychological wellbeing, quality of life, and mental health promotion. These contributions highlight the importance of using culturally sensitive and developmentally appropriate tools to capture individuals' subjective experiences and value systems.

Huang et al. validated the *Short Quality of Life Scale* (QOLS-6) among bank employees in China. Despite its brevity, confirmatory factor analysis revealed a multidimensional structure with high internal consistency. QOLS-6 scores were significantly associated with indicators of psychological distress and job satisfaction, confirming its utility as a rapid and effective tool for assessing quality of life in occupational health and broader applied contexts.

Ma et al. explored the relationship between lifestyle behaviors and subclinical depression in undergraduate students, identifying behavioral threshold through ROC analysis. Their findings support the development of concise screening tools incorporating sleep quality, sedentary behavior, and light-intensity physical activity as key factors in regulating depressive symptoms. The study highlights the potential of behavioral monitoring in mental health promotion.

Lei et al. developed and validated the *Chinese Mental Health Values Scale* (CMHVS), comprising seven culturally grounded dimensions: Expected Self, Relating to Others, Life Principles, Family, Purpose and Meaning, Achievement, and Communication. The scale demonstrated excellent reliability and convergent validity, offering a novel tool for assessing mental health attitudes and informing culturally responsive interventions.

Finally, Fernandes et al. validated the *PERMA-Profiler* for use with Brazilian adolescents, based on Seligman's wellbeing model. The five-factor structure—Positive Emotion, Engagement, Relationships, Meaning, and Accomplishment—was supported, with strong internal consistency and measurement invariance across age and gender groups. Significant associations with emotional and psychological outcomes support the *PERMA-Profiler* as a promising tool for assessing wellbeing in developmental contexts.

## Personal characteristics and coping strategies

Three studies in this Research Topic explored individual traits and psychological mechanisms that shape responses to adversity, meaning-making processes, and the management of relational and emotional demands. These contributions underscore the relevance of assessing dispositional and coping-related dimensions within the framework of Health Psychology.

Chen et al. assessed the psychometric properties of the *Existential Fulfillment Scale* (EFS) among Chinese university students. The Chinese adaptation revealed a two-factor structure—Self-acceptance and Self-breakthrough—reflecting culturally nuanced interpretations of personal growth. The scale demonstrated strong internal consistency, test-retest reliability, and meaningful associations with indicators of wellbeing and depression, supporting its use in evaluating existential aspects of psychological functioning in young adults.

Gazzellini et al. validated the *Schema Coping Inventory* (SCI) in an Italian adult sample. Grounded in schema therapy theory, the SCI measures three maladapting coping styles: surrender, avoidance, and overcompensation. CFA confirmed the original three-factor structure, and the scale showed good internal consistency. Convergent and divergent validity were supported through correlations with psychopathological symptoms and other coping constructs, positioning the SCI as a valid and reliable tool for identifying maladaptive coping patterns in both clinical and non-clinical populations.

Finally, Glavač et al. examined the *Capacity to Love Inventory* (CTL-I) in a Slovenian non-clinical sample. The original six-factor structure was replicated with satisfactory model fit and reliability. CTL-I subscales showed expected inverse correlations with dysfunctional personality traits and strong positive associations with dispositional mindfulness. These findings support the CTL-I as a valuable tool for assessing relational capacity and emotional maturity, reinforcing the connection between interpersonal functioning and psychological wellbeing.

## Conclusions

This second volume of *Measurement in health psychology, volume II* presents a diverse array of contributions that reflect the field's increasing methodological sophistication and conceptual maturity. Collectively, the studies emphasize the pivotal role of measurement in advancing both theoretical insight and clinical application within Health Psychology. Across domains including clinical assessment, psychological wellbeing, and coping mechanisms, researchers introduced and validated psychometric tools that are not only psychometrically robust but also culturally attuned and contextually appropriate.

A hallmark of this Research Topic is its consistent methodological rigor. The majority of studies employed Confirmatory Factor Analysis (CFA), with several also utilizing Exploratory Factor Analysis (EFA) and Rasch modeling. Tests for measurement invariance across gender, age, and dyadic relationships were frequently reported, reinforcing the generalizability and robustness of the proposed instruments. Notably, many contributions also focused on the development of brief yet psychometrically sound tools, addressing the growing demand for time-efficient assessments in both clinical and research settings.

In total, the 15 instruments presented in this volume expand the repertoire of tools available for assessing psychological constructs central to understanding health, illness, and adaptation. These tools offer practical solutions for measuring mental health outcomes, behavioral patterns, emotional resilience, and socio-environmental stressors, thereby contributing to a more precise and evidence-based approach in Health Psychology research and practice, in addition to relevant social impact.

This Research Topic does not intend to exhaust the field of *Measurement in health psychology, volume II*. Rather, it represents a meaningful step forward—one that we hope will inspire continued collaborative efforts to refine, validate, and disseminate assessment tools that can support the development of more effective, evidence-informed interventions and policies.
